# Global vaccine equity? Reflections, lessons, and a way forward

**DOI:** 10.1016/j.nmni.2023.101107

**Published:** 2023-02-22

**Authors:** Brianne O’Sullivan, Mohammad Yasir Essar, Mehr Muhammad Adeel Riaz, Malvikha Manoj, Marali Singaraju, Arush Lal

**Affiliations:** Department of Health Information Science, Western University, London, Ontario, Canada; Department of Global Health, McMaster University, 1280 Main Street West, Hamilton, ON, L8S 4L8, Canada; Punjab Medical College, Faisalabad, Pakistan; International Working Group for Health Systems Strengthening, United Arab Emirates; International Working Group for Health Systems Strengthening, USA; Department of Health Policy, London School of Economics and Political Science, London, UK

“Injustice anywhere is a threat to justice everywhere. We are caught in an inescapable network of mutuality, tied in a single garment of destiny. Whatever affects one directly, affects all indirectly.” - Martin Luther King Jr

In his keynote address at the 75th World Health Assembly, Dr. Tedros Adhanom Ghebreyesus, Director-General of the World Health Organization (WHO), called for a paradigm shift in global health. He spoke of the need to focus on a more comprehensive approach that includes prevention, promotion, and protection of health [1]. The COVID-19 pandemic exposed and exacerbated how vulnerable and unprepared our health systems are, and demonstrated how far we are from achieving equity in response and recovery. In order to tackle the root causes of inequity and reorient our health systems, a holistic approach to global health will be critical. It is also important to note that we already have the capacity, technology, knowledge, human power, and financing required to end this pandemic and the inequalities associated with it. However, due to resistance bourne out of capitalist approaches, geopolitical power plays, and outsized corporate interests that dictate pandemic response, our inability to meaningfully ensure health equity continues to pose an existential threat to all populations, particularly the most vulnerable and marginalized among us.

The COVID-19 pandemic reaffirmed a quasi-colonial economic order that sparked a vaccine apartheid and disproportionately marginalized the world's most vulnerable populations [2]. While high-income countries (HICs) like the United Kingdom [3] have long lifted conventional COVID-19 public health measures, the majority of populations in low-income countries (LICs) have yet to receive their first dose, let alone meet global targets [4]. At the end of 2022, only about a quarter (25%) of the African population have been fully vaccinated, and [5] nearly 5.2 million people have died as a result of vaccine apartheid [6]. The troubling reality is that the nations who proposed and advocated for the Trade-Related Aspects of Intellectual Property (TRIPS) waiver are also the ones that experienced a higher burden of morbidity and mortality from the various COVID-19 variants – largely due to imbalanced power dynamics, demonstrating the haves and have-nots in global public health. This pandemic is a wake-up call for all of us to critically examine and address the structural biases that underlie our systems and strengthen collaborative efforts to develop an inclusive system—one which cares for all and leaves no one behind.

Before the rise of Delta and Omicron variants of COVID-19, the World Trade Organization (WTO) received a request from South Africa and India in October 2020 to waive the TRIPS protections on COVID-related technologies [7]. Over 100 countries supports the proposal, which calls on active efforts to enable LMICs to manufacture affordable generic alternatives of the COVID-19 vaccine and increase global production [8]. The United States (US) officially supported a TRIPS waiver [9], but in the October 2021 TRIPS Council meeting, European Union (EU) members prevented it from being approved [10]. It is unfortunate and simply unacceptable that global health stakeholders and pharmaceutical companies in HICs are not ready to transfer the knowledge and tools that enable the production of vaccines in LMICs.

Furthermore, this trend does not end with the COVID-19 pandemic. Despite the vast inequities exposed by the COVID-19 pandemic, a similar pattern has emerged through the global vaccination response to the monkeypox outbreak, which was declared a public health emergency of international concern (PHEIC) by the WHO in July 2022 [11]. Though the virus has been endemic to West and Central Africa for decades [12], the urgency expressed in the WHO's PHEIC declaration has not been reflected in any sort of equitable global access to the vaccine. This may be attributed in part to the significant cost of the WHO-recommended monkeypox vaccine [13], the JYNNEOS vaccine, which costs approximately $110 per dose. As of August 31, 2022, the US had obtained 1.1 million doses of the JYNNEOS vaccine and ordered a further 7 million doses, approximately 80% of the available global supply and an estimated 60 available doses per monkeypox case in the US at that time [14]. Meanwhile, Africa received its first batch of mpox vaccine, which came from a donation from South Korea's government. It is worth mentioning that the majority of reported monkeypox mortalities have so far occurred in Africa. While robust monkeypox vaccination has been attributed as a key reason for plummeting rates of the virus in the US [15], the continent of Africa has yet to witness similar outcomes. In September 2022, Dr. Matshidiso Moeti, director of the African Regional Office of the WHO, shared that “Africa is still not benefiting from either monkeypox vaccines or the antiviral treatments [16]." This inequitable distribution of vaccines extends beyond Africa to the majority of LMICs globally. For example, despite having almost a tenth of reported global cases, Brazil, along with many other countries in Latin America, has also not obtained any doses of the vaccine as of September 2022 [14].

The lofty prices of this vaccine have undoubtedly contributed to the barriers LMICs are experiencing when it comes to securing doses for their populations. Rather than adopting an equitable strategy that recognizes the varying capacities of high-versus low-income countries to pay exorbitant vaccine prices, Bavarian Nordic, the manufacturer of the vaccine claimed: “We use the same approach for everyone.” Yet, equality does not equate to equity, and we are faced with yet another case of vaccine injustice [17]. Along with higher mortality rates, populations in LMICs face intersectional social, political, and structural inequities, particularly amidst the ongoing COVID-19 pandemic, which makes them more vulnerable to adverse effects of the monkeypox virus. Bavarian Nordic's emphasis on distributing health products incorrectly assumes that all populations possess the same needs and experience the same barriers, disregarding intersectional challenges experienced by LMICs during public health emergencies. In the face of another global pandemic, should the focus not be on ensuring that outbreaks everywhere are contained, regardless of financial resources? It seems that once again, profits are being prioritized over lives. The current state of monkeypox vaccine distribution indicates that “lessons learned” since the beginning of the COVID-19 pandemic have not yet been translated into action. At what point do we hold HIC leaders and pharmaceutical industries accountable for their negative and harmful actions? What is our collective accountability towards this end, whether as students, professionals, or community members?

The urgency of vaccination to respond to the COVID-19 pandemic, monkeypox, and other such health crises, as well as the significant investment required to create vaccines, highlights the need for international cooperation to create more structural and sustainable strategies than vaccine philanthropy and achieve meaningful global health equity. In December 2021, the WHO announced the formation of a new intergovernmental negotiating body (INB) to draft a new convention, treaty, or agreement under Article 19 of the WHO Constitution (which also includes the successful WHO Framework Convention on Tobacco Control) [18] with the aim of strengthening global pandemic prevention, preparedness, response, and recovery [19]. The proposed Pandemic Treaty that is currently being negotiated by member states will include both legally-binding and non legally-binding components, and will be submitted for consideration at the 77th World Health Assembly in 2024 [20]. The Pandemic Treaty presents a promising opportunity to operationalize health equity during future pandemics, specifically regarding legally-binding protocols for the equitable sharing of information, technology, and expertise across WHO member states, as well as enabling local manufacturing capacities of medications and vaccines in LMICs. Throughout the COVID-19 and monkeypox pandemics, corporate profiteering and vaccine nationalism have consistently undermined other attempts to ensure equal access to vaccines and medical treatments. This indicates that current policies and practices are not sufficient, and the Pandemic Treaty's new legally-binding measures may be the push needed to ensure equity is at the center of prevention, preparedness, response, and recovery efforts for future pandemics [21].

The world must focus on optimizing LMICs’ access to the life-saving technologies that HICs countries routinely gatekeep. In the wake of recent efforts to decolonize global health and move towards more equitable systems of health, we are yet again faced with an opportunity we seem to be failing to seize. As other crises like climate change and increasing antimicrobial resistance loom over us [22], the window of opportunity to make a drastic – but necessary -- shifts is rapidly closing. To this end, it is necessary to increase funding and support for global health initiatives in LMICs, including the transfer of technology and know-how from HICs. Additionally, we must demand transparency and accountability in global health decision-making, ensuring that diverse voices and perspectives are included in policy development. Finally, we must prioritize global health equity and solidarity in all actions, working to dismantle systems of coloniality and capitalism that have contributed to health inequities.

## Funding

No funding reported.

## Author's contributions

MYE conceptualized the topic. MYE, BOS, and MAR wrote the first draft. MM, MS wrote the second draft. AL edited the second draft and made critical comments. All authors read and approved the final draft.

## Declaration of competing interest

None declared.
